# The impact of combined age-related vision loss and dementia on the participation of older adults: A scoping review

**DOI:** 10.1371/journal.pone.0258854

**Published:** 2021-10-20

**Authors:** Colleen McGrath, Inaara Karsan, Ann Marie Corrado, Taylor Ashley Lyons, Melanie Blue

**Affiliations:** 1 School of Occupational Therapy, Western University, London, Ontario, Canada; 2 Women’s College Hospital, Toronto, Ontario, Canada; 3 London Health Sciences Centre, London, Ontario, Canada; 4 CBI Health Centre, Ottawa, Ontario, Canada; Indiana University Purdue University at Indianapolis, UNITED STATES

## Abstract

**Introduction:**

There are a growing number of older adults with combined age-related vision loss (ARVL) and dementia. Existing literature shows the pervasive impact that both diagnoses have separately on the participation of older adults, however, little is known about the societal participation of older adults with both conditions. As such, the aim of this scoping review was to explore the combined impact of ARVL and dementia on the participation of older adults, with a specific focus on highlighting strategies that help mitigate the impact of ARVL and dementia on participation.

**Methods:**

This study utilized a scoping review, informed by the framework by Arksey and O’Malley [1]. Two researchers independently ran a total of 62 search terms across four categories in six databases (PubMed, CINAHL, Scopus, Embase, Medline, PsycINFO), with an initial yield of 2,053 articles. Grey literature was also included in this scoping review and was retrieved from organizational websites, brochures, conference proceedings, and a Google Scholar search. The application of study inclusion criteria resulted in a final yield of 13 empirical studies and 10 grey literature sources.

**Results:**

Following detailed thematic analysis of the empirical and grey literature sources, four themes emerged regarding the impact of combined ARVL and dementia on the participation of older adults including: 1) Managing the pragmatic aspects of a dual diagnosis; 2) Diverse approaches to risk assessment and management; 3) Adopting a multi-disciplinary approach to facilitate care and; 4) Using compensatory strategies to facilitate participation.

**Conclusions:**

The four themes highlight the challenges older adults with these combined diagnoses experience, which limit their opportunities for meaningful participation. Given the scarcity of research on this topic, future research should identify the type of ARVL and dementia diagnoses of study participants, conduct qualitative research about the lived experiences of older adults with a dual diagnosis, and broaden the geographic scope of research.

## Introduction

Low vision refers to a permanent “loss of visual acuity (i.e., less than 6/18 but at least 3/60) or visual field (i.e., less than 20 degrees) in the better eye, not correctable by spectacles, contact lenses, or intraocular lenses” [2 p. 580]. Within industrialized countries, older adults constitute the fastest growing segment of the population with low vision, including macular degeneration, glaucoma, and diabetic retinopathy, with such conditions often collectively referred to as ARVL [3]. Dementia is an overarching term encompassing several progressive, neurodegenerative brain disorders that result in cognitive deficits [4], including Alzheimer’s disease, dementia with Lewy bodies, frontal-temporal dementia, and vascular dementia [5]. Dementia has both cognitive and neuropsychiatric clinical symptoms, including decreased memory; difficulty with new learning and problem solving; aphasia, apraxia, agnosia and executive functioning deficits; thinking, perceptual, affective, and behavioural disorders; as well as psychosis and social decline [6]. In some cases, there is a physiological link between vision loss and dementia, in that dementia impacts structures of the brain responsible for visual processing [7–9]. Similarly, a diagnosis of vision loss in adults has been linked to a higher risk of dementia [10–12]. Thus, it is often difficult to differentiate symptoms of ARVL from dementia, as one condition may mask the other.

Full participation in society is recognized by the United Nations Convention on the Rights of Persons with Disabilities as a fundamental right for all persons, including persons with disabilities [13]. Participation is broadly defined as a person’s involvement in life situations including personal care, mobility, employment, civic participation, social participation, and leisure [14]. Older adults with ARVL report that their participation is impeded primarily by mobility barriers, disabling features of the physical environment, as well as social barriers, such as stigma [15]. Further, older adults with vision loss have increased difficulty pursuing leisure interests, instrumental activities of daily living (IADL) and meaningful social interactions [15,16] which can have detrimental effects on physical, mental, and social wellbeing. Similarly, as dementia progresses, and the cognitive deficits become more debilitating, older adults may lose both their functional ability to be independent, but also the ability to perceive the need for and/or initiate typical daily activities, resulting in a decrease in meaningful participation [4,6]. Despite the known risks to participation, strategies to mitigate barriers to social participation within society are not well documented for older adults with a combined diagnosis of ARVL and dementia [17].

Existing research reports evidence of significant disturbances to visual function among older adults with dementia which may affect different aspects of visual performance and processing including depth perception, hallucinations, contrast sensitivity, object recognition, and spatial localization [18]. However, there remains a high proportion of undiagnosed older adults living with both conditions, as demonstrated in a study by Wong et al., where 66.9% of older adults with dementia were screened and diagnosed with eye diseases that they were previously unaware of [17]. Although existing research has investigated the separate impact of ARVL and dementia on participation, little is known about older adults experiencing both conditions simultaneously.

## Methods

Scoping reviews map a broad area of research, providing a picture of the main emphases and gaps within a topic area in order to convey both the breadth and depth of a field [19,20]. This scoping review was based on the framework by Arksey and O’Malley [1] which includes five key stages: 1) Identifying the research question; 2) Identifying relevant studies; 3) Study selection; 4) Charting the data, and; 5) Collating, summarizing and reporting results. The decision to conduct a scoping review, rather than a systematic review, was made because scoping reviews provide a preliminary assessment of the size and scope of the available research literature. This was necessary given the relatively broad landscape of literature focused on older adults with combined dementia and ARVL.

### Stage one: Identifying the research question

The scoping review was guided by the research question: “*How does the combined impact of age-related vision loss and dementia influence the participation of older adults*?” The authors paid particular attention to highlighting those strategies that helped to mitigate the impact of ARVL and dementia on participation.

### Stage two: Identifying relevant studies

The search strategy, including database selection, was developed by the research team in consultation with a librarian. Two researchers independently ran a total of 62 search terms across four categories in six databases including: PubMed, CINAHL, Scopus, Embase, Medline, and PsycINFO. The search was first performed in October 2019 and later updated in May 2021 in accordance with the Preferred Reporting Items for Systematic Reviews and Meta-Analyses extension for Scoping Reviews (PRISMA-ScR) Checklist [21]. Search terms were categorized into four main groupings, including “older adults”, “vision-loss”, “dementia” and “participation” (Table 1). Search terms were mapped to subject headings, and all were searched as keywords. Grey literature was included in this scoping review and was retrieved from ARVL and dementia organization websites, brochures, conference proceedings, and a Google Scholar search using the keywords “older adults” “vision-loss’, “dementia”, and “participation”. The inclusion of grey literature maximized the richness and comprehensiveness of the findings and included variability in perspectives, such as informal caregivers, healthcare providers, and older adults with combined ARVL and dementia. The researchers organized all relevant sources in the reference management system Mendeley. The reference lists of all included articles were reviewed to identify additional studies.

**Table 1:** Search terms.

Research databases searched	Search terms
• CINAHL • Embase • Medline • PsycINFO • PubMed • Scopus	• (“elder*” OR “older adults” OR “geriatrics” OR “aged” OR “older adults” OR “old* people”) AND • (“glaucoma” OR “macular degeneration” OR “diabetic retinopathy” OR “age-related vision loss” OR “vision loss” OR “vision impairment” OR “visual deficit”) AND • (“Alzheimer’s” OR “lewy body” OR “lewy bodies” OR “frontotemporal dementia” OR “mixed dementia” OR “vascular dementia” OR “demented” OR “dementia” OR “cognitive impairment”) AND • ("social inclusion" OR "social participation" OR "community participation" OR "social support" OR "social activity" OR "social integration" OR "social engagement" OR " social involvement " OR "social capital" OR "community involvement" OR "social interactions" OR "civic participation" OR "volunteer*" OR "political participation" OR "voting" OR "community mobility" OR "social network" OR "community life" OR "church" OR " religion " OR "social clubs" OR "cultural events" OR "visit family" OR "visit friends" OR "hobby" OR "shopping" OR "restaurant" OR "pub" OR "café" OR "sports" OR "library" OR "gym" OR "fitness" OR "recreation" OR "social groups" OR "community groups" OR "friendships" OR "occupational participation" OR "activity participation" OR "community")
Grey literature sources included: • Google and Google Scholar • Dementia and Sight Loss Interest Group • Royal National Institute of Blind People • College of Optometrists • Alzheimer’s Society	• older adults AND vision loss AND dementia AND participation

### Stage three: Study selection

**Fig 1:**
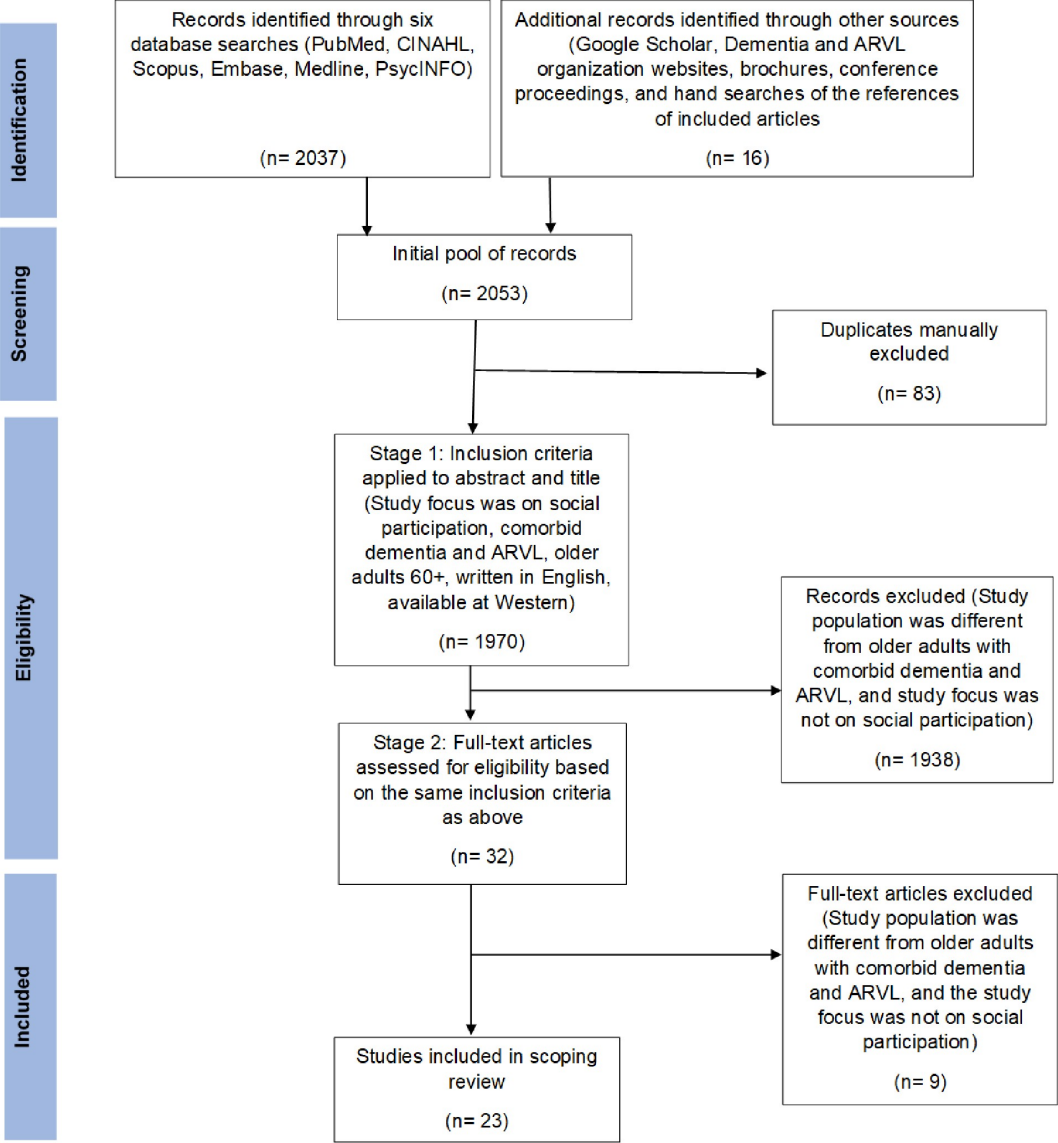
PRISMA flowchart of study selection process.

A two-stage screening process was used to assess the relevance of the studies and grey literature. The first stage included title and abstract screening followed by the second stage, which was a full-text review. Both stages of review included two independent researchers assessing all studies. A third reviewer was included if article selection discrepancies could not be managed by the two reviewers at either stage. Articles and grey literature sources were included if: a) they were published in English; b) participants were older adults aged 60+ years; c) the focus was on an outcome related to participation; d) participants had combined ARVL (macular degeneration, glaucoma, and/or diabetic retinopathy) and dementia and; e) the full text article was available through Western University. To maintain a diverse set of publications, limits were not placed on the study’s country of origin, study design, or year of publication. Details on identification, screening, and inclusion can be found in the PRISMA flow diagram (Fig 1) [21]. The application of study inclusion criteria in both stages resulted in a final yield of 13 empirical studies and 10 grey literature sources.

### Stage four: Charting the data

Co-coding of five sources was completed by the first three authors and reflexive discussions regarding approaches to coding were completed to ensure a consistent approach to coding was adopted. Once consistency in coding was achieved, the second and third authors completed a detailed article review for the remaining empirical studies and grey literature sources, capturing key information such as: author(s) or affiliated organization, year, journal/source title, location, design, aim, sample (number of participants, mean age, and diagnoses), methods of data collection and analysis, study findings/results, supporting quotes (if relevant), implications, strengths and limitations, and the domains of participation addressed. After the data was charted, a second reviewer verified the data for accuracy and updated the extraction chart, as necessary.

### Stage five: Collating, summarizing and reporting results

Thematic analysis was used to examine and record key patterns in the data [1]. Codes were initially developed independently by the first and second author, by highlighting key words or phrases used consistently in both the empirical and grey literature data extraction charts. After an exhaustive list of codes were compiled, the authors collaborated to group similar codes into larger themes through an iterative process. During this final phase of data analysis, the researchers engaged in a constant comparative method in which the similarities and differences across the empirical and grey literature were discussed and distilled. Each theme identified, addressed a barrier to participation for older adults with combined ARVL and dementia. There was agreement between the first and second authors on the themes and evidence from the empirical and grey literature to support each theme, which was mapped accordingly.

## Results

Of the 2,053 initial pool of records identified, 2,037 sources were extracted from six database searches and sixteen sources were found through other sources including hand searching the references of included articles and grey literature sources. Thirteen empirical sources and ten grey literature sources met the selection criteria and were used in the scoping review, however, one article [22] was a secondary analysis of the original data set [23].

### Characteristics of the studies selected

The empirical studies selected for this scoping review varied both in terms of research methodology (quantitative, qualitative, and mixed methods), study design (longitudinal, retrospective, cross-sectional, and case study), and data collection strategies (interviews, surveys, and focus groups) (Table 2). Of the total studies, five were qualitative, six were quantitative and two were mixed methods. The most common form of data collection was through surveys (n = 7) followed by interviews (n = 6) and focus groups (n = 2). All empirical articles were published from 2007 onwards, with the majority based in the United Kingdom (n = 6) and the United States (n = 5). The remaining articles were from Canada (n = 1) and Singapore (n = 1). All grey literature sources were based in the United Kingdom (Table 3).

**Table 2:** Demographic characteristics of empirical literature sources.

Reference	Country	Study Design (method of data collection)	Perspective Highlighted	Domain of Participation
Guthrie, D.M., et al. (2018) [37]	Canada	Quantitative. Cross-sectional study (survey)	291,824 home care clients 110,578 long term care residents	ADL; IADL; Mobility
Whitson, H.E., et al. (2014) [26]	Singapore	Quantitative (survey)	4,508 older adults	ADL; IADL; Mobility
Nyman, S.R., et al. (2017) [42]	United Kingdom	Qualitative (semi-structured interviews)	26 older adults	Leisure; ADL; IADL; Mobility; Social engagement
Dawson, A., et al. (2016) [24]	United Kingdom	Mixed methods (interviews and survey questionnaire)	10 “expert informants” interviewed 117 care professionals surveyed	Leisure; ADL; Productivity
Lawrence, V., et al. (2009) [23]	United Kingdom	Qualitative. Case-study (in-depth interviews)	17 older adults 17 family caregivers 18 healthcare professionals	Leisure; IADL; Mobility; Social engagement
Lawrence, V., & Murray, J. (2010) [39]	United Kingdom	Qualitative (in-depth interviews and focus groups)	17 care professionals	Leisure; IADL; Mobility
Evans, S.C., & Bray, J. (2016) [25]	United Kingdom	Qualitative (focus groups)	47 healthcare professionals	Leisure; Social engagement
Lawrence, V., & Murray, J. (2009) [22]	United Kingdom	Qualitative. Case study (interviews)	17 older adults 17 family carers 18 care professionals	Leisure; IADL; Mobility; Social engagement
Petrovsky, D.V., et al. (2019) [34]	United States	Quantitative. Retrospective cross-sectional descriptive study (survey)	213 nursing home residents	Leisure; IADL; Mobility; Productivity; Civic participation; Social engagement
Rovner, B.W., et al. (2009) [40]	United States	Quantitative. Longitudinal study (telephone questionnaire)	160 family members and homecare staff	Leisure; Mobility; Social engagement
Barstow, B.A., et al. (2015) [38]	United States	Mixed methods (survey, in-depth interviews, observation)	59 occupational therapists 8 older adults	Leisure; ADL; IADL; Mobility; Productivity; Social engagement
Kang, H. (2012) [35]	United States	Quantitative. Descriptive correlational study	153 older adults	Leisure; ADL; Social engagement
Whitson, H.E., et al. (2007) [36]	United States	Quantitative. Prospective cohort study (in-person survey)	3,878 older adults	ADL; IADL

**Table 3:** Demographic characteristics of grey literature sources.

Author / Organization	Year of Publication	Source Type	Country
Greasley-Adams, C., et al.	2014	Research Report	United Kingdom
Houston, A.	2016	Leaflet	United Kingdom
RNIB Scotland	n.d.	Public Resource	United Kingdom
Alzheimer’s Society	2016	Public Resource	United Kingdom
Buchanan, S., & Evers, C.	2010	Research Report	United Kingdom
College of Optometrists	2016	Research Report	United Kingdom
Dementia and Sight Loss Interest Group	2011	Research Report	United Kingdom
Dementia and Sight Loss Interest Group	2019	Research Report	United Kingdom
Skills for Care	2015	Case-Study Collection	United Kingdom
Social Care Institute for Excellence	2015	Public Resource	United Kingdom

As a result of thematic analysis of the empirical and grey literature sources, the following four themes emerged regarding the impact of combined ARVL and dementia on participation (Table 4) including: 1) Managing the pragmatic aspects of a dual diagnosis; 2) Diverse approaches to risk assessment and management; 3) Adopting a multi-disciplinary approach to facilitate care and; 4) Using compensatory strategies to facilitate participation. Although the four themes are presented separately, the authors acknowledge that the factors that shape participation are often interconnected.

**Table 4:** Identified themes in the empirical and grey literature sources.

	Theme 1: Managing the pragmatic aspects of a dual diagnosis	Theme 2: Diverse approaches to risk assessment and management	Theme 3: Adopting a multi-disciplinary approach to facilitate care	Theme 4: Using compensatory strategies to facilitate participation
Guthrie, D.M., et al. (2018) [37]	X	X	X	X
Nyman, S.R., et al. (2017) [42]	X	X	X	X
Dawson, A., et al. (2016) [24]	X	X	X	X
Whitson, H.E., et al. (2014) [26]	X		X	
Lawrence, V., et al. (2009) [23]	X	X	X	X
Lawrence, V., & Murray, J. (2010) [39]		X	X	
Evans, S.C., & Bray, J. (2016) [25]	X	X	X	X
Lawrence, V., & Murray, J. (2009) [22]	X	X	X	X
Petrovsky, D.V., et al. (2019) [34]	X		X	X
Rovner, B.W., et al. (2009) [40]		X		
Barstow, B.A., et al. (2015) [38]		X	X	X
Kang, H. (2012) [35]	X		X	
Whitson, H.E., et al. (2007) [36]	X			
Greasley-Adams, C., et al. (2014) [43]				X
Houston, A. (2016) [30]	X		X	X
RNIB Scotland				X
Alzheimer’s Society (2016) [27]	X			X
Buchanan, S. & Evers, C. (2010) [33]	X	X	X	X
College of Optometrists (2016) [41]	X		X	
Dementia and Sight Loss Interest Group (2011) [28]	X	X	X	X
Dementia and Sight Loss Interest Group (2019) [29]	X	X	X	X
Skills for Care (2015) [31]	X	X	X	X
Social Care Institute for Excellence (2015) [32]	X	X	X	X

### Theme 1: Managing the pragmatic aspects of a dual diagnosis

Managing the pragmatic aspects of a dual diagnosis relates to the symptoms and emotional challenges experienced by older adults with combined ARVL and dementia, and the resulting barriers to participation.

#### Symptom management

The order of diagnosis, meaning whether ARVL or dementia was diagnosed first, was a common factor impacting an older adult’s ability to cope [24–26]. For example, when sight loss was diagnosed first, older adults developed adaptive strategies to cope with their vision loss and navigate their physical surroundings to support their continued participation in desired activities [26]. However, significant challenges to mobility and social participation were identified when older adults with dementia subsequently developed sight loss, because their short-term memory loss made it increasingly difficult to adapt to new routines [24,25].

Disorientation among older adults with a dual diagnosis impacted participation, due to a lack of anchoring visual cues and a greater reliance on caregiver support [22,27–32]. An older adult with ARVL and dementia described their experience with disorientation by stating: “I was with my cousin having lunch and he went off to go back home and I was in the garden and I just could not place myself. And I said to, in a loud voice, ‘I hope somebody will come and fetch me for tea this afternoon, I don’t think I know where I am’. I just didn’t know where I was and you know that was really frightening you know. I thought I could fall down and break a leg” [23 p. 514]. Both empirical and grey literature sources also found that experiences of disorientation frequently resulted in emotional distress that manifested in agitated behaviors, such as abruptly wandering off and pacing, which significantly impacted participation in desired activities [22,23,27,29,32].

Managing visual hallucinations was a challenging symptom described in two empirical studies and five grey literature sources [22,23,27,29,30,32,33]. For example, Lawrence et al. [23] found that 7 out of 19 participants with ARVL and dementia experienced visual hallucinations, which increased feelings of distress and disorientation [22]. Adding to this challenge, family members were often not trained on how to properly address the visual hallucinations experienced by their loved ones [23]. Although sources were unclear regarding the best solution, the grey literature highlighted management techniques including providing verbal reassurance, adopting a non-confrontational communication style, and distraction/re-direction [27,29] as strategies to help manage visual hallucinations and promote the participation of older adults with a dual diagnosis.

#### Challenges to emotional wellbeing

Difficulty accepting multiple losses, impacted older adults’ emotional wellbeing and negatively affected participation [22,23,31,33]. Specifically, older adults with combined ARVL and dementia struggled with the threat of losing their sense of identity, which led to the employment of self-protective strategies, such as denial [22]. Additionally, it was reported that when individuals experienced one form of loss, it was more difficult to accept a second loss [34]. For example, the Dementia and Sight Loss Interest Group [28] demonstrates how the layering of diagnoses can create barriers to meaningful participation: “It’s so hard for Dad to do the things he liked to do. It’s not just the effect of macular disease and his poor central vision, but the confusion and loss of memory because of the dementia makes everything twice as difficult for him; you can see him just giving up” [28 p. 3].

Four empirical studies and four grey literature sources identified loneliness, and subsequent depression, as common emotional responses that impacted the participation of older adults with combined ARVL and dementia [22,23,28,31–33,35,36]. For example, Guthrie et al. [37] reported that 15.4% of participants in a homecare setting, with cognitive and vision impairment, self-reported feeling lonely while an additional 28.2% (in homecare) and 32.8% (in long-term care) reported symptoms of depression which invariably impacted the desire to participate in meaningful activities.

### Theme 2: Diverse approaches to risk assessment and management

The different approaches to assessing and managing risk adopted by caregivers, such as an overcautious approach versus a person-centered approach, impacted the participation of older adults with combined ARVL and dementia.

#### Taking an overcautious approach to risk management

A preoccupation with safety and overestimating risk can lead to caregivers taking an overcautious approach when working with older adults with combined ARVL and dementia. In turn, limitations may be placed on older adults’ participation in valued activities, resulting in a loss of independence and a greater reliance on caregivers to support continued activity engagement [23,25,28,38]. Further, an overcautious approach was observed when formal caregivers did not understand the needs or wants of the older adult or when they felt inadequately trained to support an older adult with combined ARVL and dementia [38]. For instance, an occupational therapist in Lawrence et al. [23 p. 514] stated: “You see them being herded about, put in wheelchairs even though they can walk but it’s safer for the nursing home to put them in a wheelchair and push them from a to b”. This overcautious approach, largely stemming from a desire to maintain safety, impacted the participation of older adults with a dual diagnosis in meaningful activities that were perceived as too “risky” by formal caregivers. Stemming from a desire to protect older adults with a dual diagnosis, conflict in the caregiving relationship often occurred. For example, family members often restricted their loved one from participating in valued activities, such as woodworking or neighbourhood walks, because they felt the older adult lacked the necessary judgement or insight [23,28]. A quote from the Dementia and Sight Loss Interest Group highlighted this: “Dad loved his workshop, but his poor sight and confusion meant we were too worried to let him use it. We started to lock it- and that made him so angry. Now we have worked out that when he helps with jobs in the house and garden he is safer than we thought- and he is happier” [28 p. 1].

#### Adopting a person-centered approach to risk management

Five studies discussed an alternative approach to risk management, that of taking a person-centered approach which requires formal caregivers to work collaboratively with older adults to understand their individual needs, abilities, and wishes in an effort to support their engagement in valued activities [22,24,34,37,39,40]. A person-centered approach minimizes the risk of formal caregivers making faulty assumptions about the older adult’s competence and instead focuses on preserving the older adults’ integrity and promoting the development of meaningful social connections [39].

### Theme 3: Adopting a multi-disciplinary approach to care

Although there was minimal evidence of collaboration between dementia services and low vision rehabilitation, seven empirical studies and four grey literature sources acknowledged that increased collaboration would be beneficial to achieve a holistic understanding of the older adult’s capabilities and promote societal participation [22–25,28,31,33,35,39,41,42]. A multi-disciplinary approach could be achieved by implementing joint training initiatives and collaborating on client assessments.

#### Implementing joint training and education initiatives

As a strategy to support the participation of older adults with a dual diagnosis of ARVL and dementia, the literature addressed the need to improve training and education for low vision rehabilitation and dementia services [22,24,25,39,42], thereby providing a more holistic approach to care. Many factors contributed to the inadequate training of staff working with this population, including formal caregivers failing to recognize the roles of other healthcare providers or how to refer to those services, as well as pragmatic considerations including a lack of personnel, time, and budget necessary to facilitate joint training and educational programming [24,39].

Five empirical articles and four grey literature sources acknowledged that joint training for low vision rehabilitation and dementia services should focus on raising awareness of the functional limitations imposed by each diagnosis and understanding how to promote the autonomy of older adults with a dual diagnosis in an effort to support engagement in activity [22,24,25,29,31,39,41–43]. Several training opportunities were recommended including presentations by both low vision and dementia services, instructional videos, professional shadowing opportunities, and case studies to help professionals develop insight into the experiences of older adults with a dual diagnosis [24]. Along with training formal caregivers, resources for families and an increased quality of post-diagnosis support also needs to be provided [25].

#### Conducting joint client assessments

The current model of care for clients with combined ARVL and dementia involves a separate assessment of each condition and their associated impacts on client function and participation; however, concurrent assessments would more accurately identify the root cause of common underlying symptomology, including disorientation and visual hallucinations [39]. As an example, a sight loss professional in a study by Evans and Bray, explained: “A lot of (symptoms) sometimes (are) put down to the dementia, when actually it might be, some of it might be the sight loss" [25 p. 95]. Unfortunately, when underlying symptomology is misdiagnosed, it can delay treatment which is problematic given that support initiated early after diagnosis is necessary in order to facilitate client education, training, and the provision of adaptive strategies and assistive technology [25,28,29,41] which are intervention strategies deemed necessary to support the participation of older adults with a dual diagnosis.

Collaboration in service delivery increases confidence among formal caregivers, as they no longer have to rely on their limited knowledge of a condition that may be outside their area of clinical expertise [23,29,35,39,41]. For example, a rehabilitation worker in a study by Lawrence and Murray explained the benefit of joint working: "I think actually there is enormous learning in joint working as well you know when somebody says well I would be looking at this in this way, and then I could say well I would look at it this way, and this can be dovetailed together" [39 p. 478].

### Theme 4: Using compensatory strategies to facilitate participation

Older adults with combined ARVL and dementia require compensatory strategies to support participation including optimizing the physical environment, enhancing communication interactions, adopting a multisensory approach, and utilizing assistive technology.

#### Optimizing the physical environment

Five empirical studies and nine grey literature sources discussed how optimizing the physical environment for an older adult with combined ARVL and dementia supported navigation as well as provided increased opportunities for participation [24,25,27–34,38,42,43]. Consistent recommendations included: adjusting lighting types and levels to reduce shadows and glare, maintaining a familiar living environment by either living at home or bringing furnishings to a new living residence, consistent signage with large block font and clear colour contrast, and minimizing visual and physical obstacles in the home to reduce falls risk [29].

#### Enhancing communication interactions

In three empirical studies and three grey literature sources, improved communication between both formal and informal caregivers and older adults with a dual diagnosis supported continued activity engagement [22–24,29,32,33]. Optimal communication strategies identified included: using a clear and soft tone of voice, speaking to the client one-on-one in a familiar environment, ensuring caregiver consistency so that rapport could be established, and providing simple step-by-step instructions to reduce disorientation, distress, and agitation [23,24,28,32,42].

#### Adopting a multisensory approach

In the absence of anchoring visual cues, utilizing a multisensory approach is necessary to support continued participation in meaningful activity. Specifically, Dawson et al. discussed the shift from a visual learning platform towards the use of other senses (auditory, touch, and olfactory) when engaging in new activities: “Somebody with dementia…will watch closely and try and mirror what other people are doing to make sense of what is going on around them because instructions might be too complex. If they cannot see visually what to do and how to be cued in, then they really are reliant on that guiding voice and that guiding touch” [24 p. 47]. An activity like gardening, for example, adopts a multisensory approach with its distinct smell and repetitive physical movements [23,25,34,40].

#### Assistive technology

Three grey literature sources [28–30] detailed the types of assistive technologies commonly used by older adults with combined ARVL and dementia to support their participation in meaningful activity including: automatic lights, audio labels, talking books, white cane, large button television remotes and telephones, large print clocks, as well as large print playing cards. Although the evidence suggested that the use of assistive technologies can promote independence and participation among older adults with the combined diagnoses, there are several barriers to use [23,42]. For example, in addition to being expensive, assistive technology often depends on the reliance of visual cues and memory to learn how to use it, which can limit the effectiveness of assistive technologies for older adults with ARVL and dementia [42]. However, barriers to assistive technology use in this population can be partially overcome by implementing audio cues and a user-friendly interface [42]. Further, studies concluded that early introduction of technology, in an effort to facilitate learning and routine, can benefit the participation engagement of older adults with combined ARVL and dementia [25,42].

## Discussion

This scoping review examined 23 empirical or grey literature sources that examined the influence of combined ARVL and dementia on the participation of older adults. Without any exclusion criterion applied regarding year of publication, all literature included in this scoping review were published from 2007 onwards, suggesting that interest in studying the challenges associated with a dual diagnosis of ARVL and dementia is relatively new. Additionally, all empirical and grey literature sources were published within developed nations including the United States, United Kingdom, Canada, and Singapore suggesting a lack of representation in the existing literature from the developing world.

The first theme highlighted the importance of addressing the underlying symptomology and emotional challenges of combined ARVL and dementia. Specifically, results identified the importance of early diagnosis and treatment, particularly when vision loss was diagnosed first, as the literature suggests that older adults who receive an ARVL diagnosis prior to a dementia diagnosis are better positioned to develop adaptive strategies, such as using assistive technology to support ongoing participation [24,25]. The review findings also identified the importance of caregivers acknowledging the emotional impact of a combined diagnosis of ARVL and dementia, including the resulting sense of identity loss. These findings clearly demonstrate the importance of older adults with a dual diagnosis and their caregivers being linked to appropriate services (ideally using a multidisciplinary approach), to help develop healthy coping strategies to facilitate meaningful participation. One such resource is peer support groups. The broader gerontological literature has shown that peer support groups, for both older adults and caregivers, can reduce loneliness and isolation among older adults, while further reducing caregiver stress [44]. In fact, peer support has been routinely used as a post-diagnostic intervention among older adults with varying conditions including ARVL, dementia, diabetes, and chronic low back pain [44–47]. The findings make clear the need for healthcare providers to understand the symptomology, emotional challenges of a dual diagnosis, and the resources/services available in their community, such as peer support groups, such that appropriate and timely referrals can be made.

The second theme highlighted formal and informal caregivers’ preoccupation with safety, which often restricted the independence and participation in meaningful activity of older adults with ARVL and dementia. An overcautious approach was frequently taken when formal caregivers were poorly trained regarding the functional needs of older adults with a dual diagnosis or were pre-occupied with managing older adults’ participation in activities perceived as “risky” [22–24,38]. To maintain older adults’ participation in valued activities and preserve their sense of identity, many studies suggested implementing a person-centered approach to risk management. Adopting a person-centered approach ensures that older adults’ needs, wishes, and abilities drive the rehabilitation process rather than a risk management perspective that serves to limit participation. A systematic review by Kim and Park [48] assessed the implications of person-centered care on people with dementia and found that this approach reduced feelings of agitation and aggression, as well as improved quality of life. Healthcare providers whose practices are grounded in person-centred care, such as occupational therapy, are well positioned to integrate such principles into their rehabilitation plans with older adults with combined ARVL and dementia.

The third theme of this scoping review discussed how the lack of collaboration between vision rehabilitation and dementia services are a barrier to providing quality care for older adults with combined ARVL and dementia. Moving forward, adopting a multi-disciplinary approach would allow for the more holistic treatment of older adults with ARVL and dementia, as formal caregivers would better understand each other’s scope of practice, when/how to refer patients to other services, and basic information regarding the diagnoses to differentiate the symptoms that may result from vision loss versus dementia. A collaborative approach to care, such as by using a joint clinical assessment, could more accurately ascertain the origin of underlying symptomology, which should result in better health outcomes. The World Health Organization (WHO) highlighted the relationship between training and a collaborative approach to care in their *Framework for Action on Interprofessional Education and Collaborative Practice*. The document encourages interprofessional education be part of the training of healthcare practitioners in order to reach the goal of interprofessional collaborative practice [49]. Achieving this goal, however, requires a commitment to include content regarding the challenges of a dual diagnosis within professional training programs (such as occupational therapy, physiotherapy, recreational therapy, optometry, etc.) as well as the provision of ongoing post-graduate learning opportunities through workshops, conferences, or webinars.

Lastly, the fourth theme of this scoping review identified how compensatory strategies are beneficial to support the participation of older adults with ARVL and dementia. For example, environmental modifications such as improved lighting, colour contrast, removing obstacles, and maintaining a sense of familiarity were all posited as helpful to create more vision and dementia friendly environments [23,25,34,38,42]. Such research is necessary as it moves the focus away from ‘person-fixing’ towards addressing those environmental conditions that serve to restrict participation for older adults with ARVL and dementia. Moving forward, training on these inclusive design principles should be provided across healthcare programs including occupational therapy and low vision rehabilitation, to name a few. Creating inclusive spaces should also consider the social environment. For example, campaigns to educate the broader public about the needs of this population and breaking down the stigma that is too often associated with both ARVL [50–52] and dementia [53–55], are necessary to best support older adults with this dual diagnosis. Another significant finding of this scoping review, as it relates to compensatory strategies, is the promotion of autonomy through the provision of assistive technology for older adults with a dual diagnosis. Such technology needs to be prescribed early by healthcare providers, with multiple modes of instruction used to support training. For example, instructions could vary from step-by-step written instructions with large font and associated pictograms, one-on-one hands-on training in the environment where the technology will be used, or videos that could be re-watched as necessary, all of which are training models supported by existing gerontological literature to maximize learning potential [24,42,56,57]. In many studies, cost was highlighted as a significant barrier to assistive technology use [25,42,57]. Therefore, healthcare providers prescribing assistive technology, such as occupational therapists, should provide older adults with opportunities to trial devices prior to purchase, particularly given the evolving nature of their visual and cognitive needs.

### Study limitations

There are methodological limitations of this scoping review that must be considered. First, no quality criteria were applied during the article selection process. Although that is not a methodological requirement of scoping reviews, it does place limits on the author’s ability to comment on the robustness and rigour of the included studies, thereby limiting the confidence that can be placed in the conclusions drawn from the included studies. However, considering the already limited number of studies available, conducting a quality appraisal of the literature may have significantly reduced the number of articles included in this scoping review.

A strength of this scoping review was the inclusion of grey literature, which helped to provide clinical relevance to the findings, however, many of the empirical articles and grey literature sources were written by the same authors, with some pulling results from the same data sets and all sources were from the developed world, including the United Kingdom, United States, Canada, and Singapore. This may have been due to an inclusion criterion, in this scoping review, that all research be available in English. This finding is not particularly surprising given the specific nature of the research topic; however, it does place limits on the generalizability of the findings. Moving forward, research from the developing world would greatly expand our contextual understandings of the combined impact of ARVL and dementia on participation.

A further methodological limit of this scoping review is that the authors did not complete the sixth, and optional, consultation phase. By conducting a consultation phase, it would have helped to validate the findings within the experiences of those living with, or caring for, individuals with combined ARVL and dementia.

### Future research

The limitations of the scoping review point to important areas for future research. As a starting point, additional research in this area is needed considering that only 23 articles were included in the scoping review, all of which were published within the last 15 years.

More specifically, few articles included in this scoping review specified the type of ARVL or dementia of the study participants. Information regarding the type, duration, and severity of diagnoses, as well as the order of onset, could be used in future quantitative studies to map demographic variables to their impact on participation levels. Further, more qualitative research should be conducted, specifically including older adults with a dual diagnosis, to explore their lived experiences. This is particularly relevant given that, in this review, only four studies focused solely on the older adult’s perspective with most articles relying on a proxy, such as formal or informal caregivers, to relay the participants experiences. Moving forward, it would also be valuable to expand the geographic scope of future studies, with a particular focus on the developing world, as an overwhelming majority of both empirical (N = 6) and grey literature sources (N = 7) were based in the United Kingdom, which limits the applicability of findings to a global scale. Lastly, given that the consultation phase of the scoping review process was not completed as part of this study, the authors propose carrying out this stage as the next phase of research to ground the review findings in lived experience.

## Conclusion

Despite the growing presence of older adults aging with combined ARVL and dementia, there is relatively little known about the impact on societal participation of older adults aging with both conditions. This scoping review highlighted four themes that unpack the challenges older adults with combined ARVL and dementia experience, which limit their opportunities for meaningful participation. The scoping review further posited strategies to help mitigate the impact of a dual diagnosis on participation including supporting better symptom management, integrating a person-centred approach to risk management, adopting a multi-disciplinary approach to care, and relying on compensatory strategies (including environmental modification, enhancing communication, adopting a multisensory approach, and using assistive technology) to support optimal participation.

This review demonstrated that representation in research matters. First, the research in this field is limited, albeit growing, with all included sources published within the last 15 years, suggesting that more research is needed. More specifically, this scoping review demonstrated that research that prioritizes the voices of older adults with combined ARVL and dementia, and not their family or healthcare providers acting as a proxy, are needed to better understand the lived experience of aging with this dual diagnosis. Lastly, this work demonstrated a lack of representation in the existing literature from the developing world which points to the importance of future work that pushes the contextual boundaries of this field.

## Supporting information

S1 File PRISMA-ScR checklist. (PDF)
